# Low-Cost Cellulase-Hemicellulase Mixture Secreted by *Trichoderma harzianum* EM0925 with Complete Saccharification Efficacy of Lignocellulose

**DOI:** 10.3390/ijms21020371

**Published:** 2020-01-07

**Authors:** Yu Zhang, Jinshui Yang, Lijin Luo, Entao Wang, Ruonan Wang, Liang Liu, Jiawen Liu, Hongli Yuan

**Affiliations:** 1 State Key Laboratory of Agrobiotechnology and Key Laboratory of Soil Microbiology, Ministry of Agriculture, College of Biological Sciences, China Agricultural University, Beijing 100193, China; B20173020090@cau.edu.cn (Y.Z.); yangjsh1999@cau.edu.cn (J.Y.); rnwang@cau.edu.cn (R.W.); B20163020078@cau.edu.cn (L.L.); SZ20143020044@cau.edu.cn (J.L.); 2 Fujian Institute of Microbiology, Fuzhou 350007, China; luolijin@sina.com; 3 Departamento de Microbiología, Escuela Nacional de Ciencias Biológicas, Instituto Politécnico Nacional, Mexico City 11340, Mexico; entaowang@yahoo.com.mx

**Keywords:** glycoside hydrolyase, *Trichoderma harzianum*, complete saccharification, lignocellulose

## Abstract

Fermentable sugars are important intermediate products in the conversion of lignocellulosic biomass to biofuels and other value-added bio-products. The main bottlenecks limiting the production of fermentable sugars from lignocellulosic biomass are the high cost and the low saccharification efficiency of degradation enzymes. Herein, we report the secretome of *Trichoderma harzianum* EM0925 under induction of lignocellulose. Numerously and quantitatively balanced cellulases and hemicellulases, especially high levels of glycosidases, could be secreted by *T. harzianum* EM0925. Compared with the commercial enzyme preparations, the *T. harzianum* EM0925 enzyme cocktail presented significantly higher lignocellulolytic enzyme activities and hydrolysis efficiency against lignocellulosic biomass. Moreover, 100% yields of glucose and xylose were obtained simultaneously from ultrafine grinding and alkali pretreated corn stover. These findings demonstrate a natural cellulases and hemicellulases mixture for complete conversion of biomass polysaccharide, suggesting *T. harzianum* EM0925 enzymes have great potential for industrial applications.

## 1. Introduction

Recently, bio-based industries have developed rapidly, and potential bio-based products including ethanol, aldehydes, organic acids, polyhydric alcohols, and other bio-chemicals and biomaterials have attracted a lot of attention. As the most important intermediate compounds of biological and chemical transformation from biomass, fermentable sugars (mainly glucose and xylose) are crucial for the production of downstream products [1,2]. Due to the structural complexity of lignocellulose, complete enzymatic deconstruction requires the synergistic action of cellulases, hemicellulases, and ligninases [3]. In order to obtain high yield of glucose from cellulose, endoglucanase, cellobiohydrolase, and β-glucosidase are required to work together. Endoglucanases randomly cleave the internal *O*-glycosidic bonds and produce glucan chains with different lengths, cellobiohydrolases attack the reducing and non-reducing ends of cellulose to release β-cellobiose, and β-glucosidases hydrolyze the terminal non-reducing β-d-glucosyl residues into glucose. These enzymes can be produced by many fungi (like *Aspergillus* and *Trichoderma*) and bacteria (like *Cellulomonas* and *Clostridium*) [2,3]. In any case, the rapid and complete conversion of cellulose is the limiting step during the process of bio-refinery [4].

Currently, *Trichoderma reesei* has been commonly used as a cellulase producer. Although two exoglucanases (CBH I and CBH II), eight endoglucanases (EGI-EGVIII), and seven β-glucosidase (BG I-BG VII) are produced by this fungus, the fairly low protein abundance (<1%) of β-glucosidase in the extracellular proteome limited the effective conversion of cellubiose to glucose [5]. The total cellulase activities of commercial preparations Viscozyme L and Celluclast 1.5L were as high as 33 and 95.2 FPU/mL, but the β-glucosidase activity was only 0.2 and 0.3 U/mL, respectively [6]. When β-glucosidase preparation Novezyme 188 was supplied to Celluclast 1.5L, glucose yield from sugarcane bagasse increased 1.5-fold than that of Celluclast 1.5L alone [7]. In addition, higher content of cellobiohydrolase in *Trichoderma reesei* extracellular proteome was detected, which caused the accumulation of cellobiose on account of the deficient amount of β-glucosidase, and then gave rise to severe product feedback inhibition of endoglucanase and cellobiohydrolase [8]. Some researchers found that hemicellulases, oxidoreductases, non-hydrolytic proteins and other auxiliary enzymes synergize to achieve an efficient enzymatic hydrolysis of cellulose [2]. Hemicellulase and pectinase could degrade the non-cellulose polysaccharide that covered cellulose and in turn increase the cellulose hydrolysis efficiency, and this synergistic effect could give rise to the cost reduction in deconstruction of lignocellulose [9]. Goldbeck et al. [10] found that when xylanase was added to the commercial cellulase preparation Accellerase 1500, the glucose yield of dilute acid pretreated sugarcane bagasse presented a 1.4-fold increase than that of Accellerase 1500 alone. Furthermore, hemicellulose hydrolysis products (xylose, arabinose, mannose, and galactose etc.) also have great potential in food and feed industry applications. Therefore, in terms of the whole component utilization of plant cell wall polysaccharides, not only lack of β-glucosidase restricted the efficient conversion of cellulose, the lack of hemicellulase in *Trichoderma reesei* extracellular proteome also made it difficult to utilize hemicellulose [11]. Hemicellulase activity of commercial cellulase production model strains *T. reesei* QM6a and *T. reesei* QM9414 has been determined by Li et al. [12], in which the xylanase activity was 5.42 and 1.27 U/mL, while xylosidase activity was only 0.4 and 0.005 U/mL, respectively. Recent studies showed that extracellular enzymes from *Penicillium* species perform better than cellulases from *Trichoderma* sp. On account of the higher hemicellulases activities of enzyme mixture secreted by *Penicillium* sp., greater fermentable sugar yield was obtained [13,14]. Yang et al. [15] found that when 50% of the commercial cellulase was replaced by *Penicillium chrysogenum* enzyme cocktail, release of reducing sugars was 78.6% higher than that with the cellulase alone, while the glucan and xylan conversion was increased by 37% and 106%, respectively. Additionally, the hydrolysis efficiency of cellulase increased more with the addition of multi-component hemicellulases cocktail than with xylanase alone. The artificial enzyme cocktail of pectinase, xylanase, arabinofuranosidase, acetyl xylan esterase, and ferulic acid esterase presented higher efficiency of bagasse degradation than the single component [16]. The appropriate dosage of auxiliary enzymes for efficient conversion of different lignocellulosic biomass is still uncertain. Therefore, exploring novel lignocellulosic degrading strains with complete and balanced enzyme cocktail for efficiently saccharification of lignocellulose is necessary for industrial enzyme preparation production. 

Production of extracellular enzymes with high cellulase activity has been proved in members of *Trichoderma* [17]. *T. reesei* Rut-C30 mainly expresses cellulases including cellobiohydrolase and endoglucanase with a total abundance of 90–95% of the extracellular proteins [18]. Compared with the industrial cellulase producer *T. reesei*, *Trichoderma harzianum* harbors more comprehensive lignocellulosic degrading enzyme encoding genes in its genome [19]. To be specific, a total of 42 cellulase genes and 24 hemicellulase genes were annotated in genome of *T. harzianum* T6776, which were 1.5 and 1.7-fold of those of *T. reesei* Rut-C30 [20]. Meanwhile, the expression levels of xylanase, mannase, and various glycosidases of *T. harzianum* was significantly higher than that in *T. reesei* [19,21]. Compared with *T. reesei*, some *T. harzianum* strains produced a cellulolytic complex with higher β-glucosidase and endoglucanases activities, and the xylanase activity of some *T. harzianum* strains was higher than that of *T. reesei* [22,23,24]. However, efficiency of simultaneous cellulose and hemicellulose hydrolysis by the secretome of *T. harzianum* was still low [23,25]. Lignocellulose degrading enzymes in microbes are induced by substrates, and the low-cost carbon source and culture conditions had a great influence on the enzyme secretion and composition [26]. Nevertheless, the mechanism of lignocellulolytic enzyme inducing was still unclear [27]. So, searching and exploring highly effective enzyme systems for efficiently converting the whole component of plant cell wall polysaccharides is expected, and the comprehensive understanding of secretomes in the related microbes could help the development of efficiently tailor-made lignocellulolytic enzyme cocktails in vitro [28]. Isolated in our laboratory, *T. harzianum* EM0925 could secret high levels of cellulase and hemicellulase simultaneously, and its extracellular enzyme cocktail showed strong ability of lignocellulose degradation. The enzyme cocktail of *T. harzianum* EM0925 contained a great amount of complete lignocellulolytic enzymes as revealed by proteome analysis under the optimal inducing condition. Aiming at analyzing the degradation mechanism of *T. harzianum* EM0925, we performed the present study. The results provide a basis for tailor-made preparation of low-cost enzymes to effectively degrade the whole component of plant cell wall polysaccharides.

## 2. Results

### 2.1. Substrate Selection for Lignocellulosic Enzyme Production by T. Harzianum EM0925

**Figure 1:**
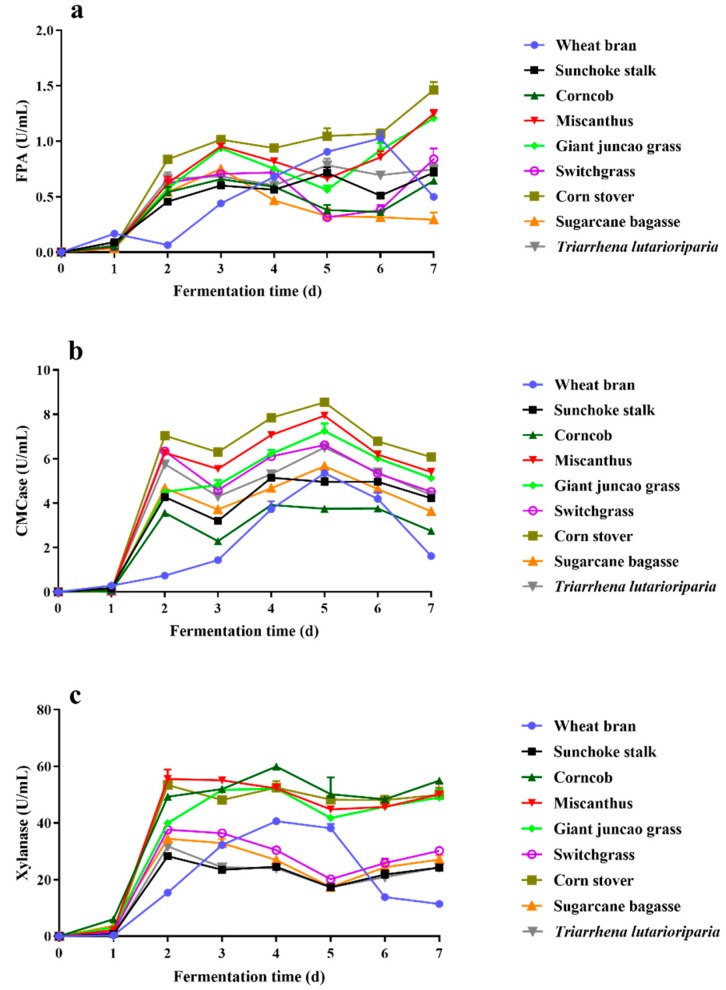
Activities of cellulase and hemicellulase secreted by *T. harzianum* EM0925 induced by different kinds of lignocellulosic biomass. (**a**) FPA in cultures; (**b**) CMCase activities in cultures; (**c**) hemicellulases activities in cultures.

In order to prepare low-cost lignocellulolytic enzymes cocktails, different kinds of lignocellulosic substrates were used separately as the carbon source for *T. harzianum* EM0925, including wheat bran, sunchoke (*Jerusalem artichoke*) stalks, corncob, miscanthus, giant juncao grass, switchgrass, corn stover, sugarcane bagasse, and straw of *Triarrhena lutarioriparia*. Measurement of the extracellular cellulase and xylanase revealed that the highest filter paper activity (1.54 U/mL) was induced by corn stover after seven days of cultivation (Figure 1). Simultaneously, high levels of endoglucanase activity (8.61 U/mL) and xylanase activity (54 U/mL) were induced (Figure 1), in which the xylanase activity was 91% of the maximum value (59.9 U/mL) detected in the culture with corncob after seven days of cultivation. In addition, when corn stover was used, rapid enzyme production capacity was observed in *T. harzianum* EM0925. The filter paper activity reached more than 70% of the maximum value, while the endoglucanase and xylanase activities reached more than 80% of their maximum values after two days of fermentation. Therefore, corn stover was selected as the optimal substrate for *T. harzianum* EM0925 to prepare the lignocellulolytic enzyme cocktail (EM0925) in further study.

### 2.2. Enzyme Activities of Enzyme Cocktail EM0925

Enzyme activities of commercial preparations (C 9748 and Celluclast 1.5L) and EM0925 (Enzyme preparation of *T. harzianum* EM0925) were measured by using the model substrates. The three enzyme preparations showed significant differences in their specific activities of cellulases, including endoglucanase, cellobiohydrolase, and β-glucosidase. The endoglucanase activity of EM0925 was only 56.9% of C 9748, and the cellobiohydrolase activity showed no significant difference with that of Celluclast 1.5L. Most notably, EM0925 displayed the highest level of β-glucosidase activity, which was 311 and 2.9 folds of that in C 9748 and Celluclast 1.5L, respectively. In addition, EM0925 displayed significantly higher hemicellulases activities than the two commercial preparations. The xylanase specific activitiy of EM0925 was 310.70 U/mg, which was 29.1 folds higher than that of Celluclast 1.5L. Xylosidase and arabinofuranosidase activities were as high as 203.60 and 11.72 U/mg, which was 333.8 and 37.8 folds higher than that of commercial enzyme preparations C 9748 and Celluclast 1.5L, respectively. Furthermore, amylase activities of EM0925 and C 9748 were 3.92 and 0.3 U/mg, respectively, and it was not detected in Celluclast 1.5L. EM0925 contained a more complete lignocellulosic enzyme system than the commercial enzyme preparations. It presented high levels of glycosidases that were necessary for cellulose and hemicellulose degradation, and showed a broader prospect in fermentable monosaccharide production from complex polysaccharide compounds (Table 1).

### 2.3. Extracellular Enzymes in the Proteome of T. harzianum EM0925

**Figure 2:**
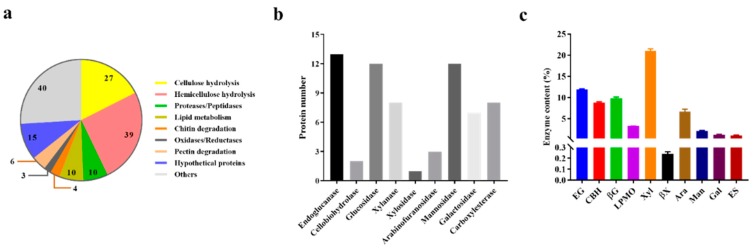
Functional classification of enzymes secreted by *T. harzianum* EM0925. (**a**) Functional classification of *T. harzianum* EM0925 enzyme cocktail; (**b**) numbers of different lignocellulosic enzyme in proteome of *T. harzianum* EM0925. (**c**) Abundance of lignocellulose degradading enzymes in the proteome of *T. harzianum* EM0925. EG, Endoglucanase; CBH, cellobiohydrolase; βG, β-glucosidase; LPMO, lytic polysaccharide monooxygenase; Xyl, xylanase; βX, β-xylosidase; Ara, arabinofuranosidase; Man, mannosidase; Gal, galactosidases; ES, carboxylesterase.

By using the label free quantitative proteomic approach, a total of 154 proteins were identified in the *T. harzianum* EM0925 secretome, which were common in all the three biological replicates. The predicted proteins in the secretome of *T. harzianum* EM0925 were grouped according to their biological function annotated in Pfam and dbCAN database. The 81 detected CAZymes contained 27 cellulases covering a complete cellulolytic enzyme system with 13 endoglucanases (GH5, GH7, GH16, GH17, GH30, GH55, GH64, GH81), 2 cellobiohydrolases (GH6, GH7), and 12 glucosidases (GH3, GH15, GH16, GH55) (Figure 2a, Figure S1). The detection of 39 hemicellulases revealed that this kind of enzymes was more abundant than the cellulases in *T. harzianum* EM0925 secretome, which included 8 xylanases (GH11, GH30), 1 xylosidases (GH3), 3 arabinofuranosidases (GH54, GH127), 7 galactosidases (GH2, GH22, GH27, GH30), 11 mannosidases (GH2, GH47, GH92, GH125), 1 mananase (GH26), and 8 carboxylesterases (CE1, CE5, CE10) (Figure 2b, Figure S1).

**Figure 3:**
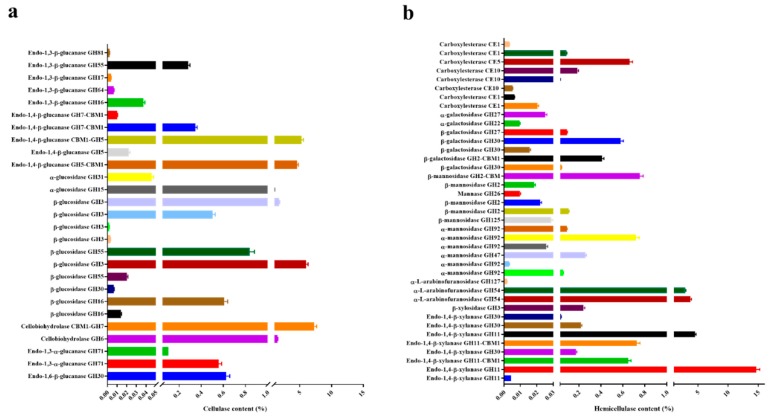
Cellulase and hemicellulase abundance in the proteome of *T. harzianum* EM0925. (**a**) Cellulase content in the proteome of *T. harzianum* EM0925; (**b**) hemicellulase content in the proteome of *T. harzianum* EM0925.

The quantitative proteomic data showed high ratios in the protein abundance of a core set of glycoside hydrolases secreted by *T. harzianum* EM0925, including total cellulase abundance of 31.5% and total hemicellulase abundance of 32.2% (Figure 2c). Cellulases, including endoglucanases, cellobiohydrolases and glucosidases accounted for 11.85%, 8.8%, and 9.76% of the total proteins, respectively (Figure 2c). Among the secreted cellulases and hemicellulases, some proteins in these sets took up a great proportion. For instance, the two GH5 endogluananses accounted for 4.54% and 5.30% of the total proteins, respectively, and both contained a CBM domain at each of the C and N ends of the protein. Cellobiohydrolases I (GH7) and cellobiohydrolases II (GH6) accounted for 7.30% and 1.50% of total protein abundance, respectively. Two GH3 β-glucosidases accounted for an outstanding proportion among the multiple glucosidases, with abundances of 6.00% and 1.76% (Figure 3a). Multiple abundant hemicellulases were also detected, including xylanase (21.01%), xylosidases (0.24%), arabinofuranosidases (6.66%), galactosidases (2.1%), mannosidases (1.18%), and carboxylesterase (1.01%). It was noteworthy that two highly expressed xylanases were relatively abundant in the secretome, with an abundance index of 14.69% and 4.51%, and accounted for 91.4% of the total xylanase abundance. A lytic polysaccharide monooxygenase (LPMO, AA9) secreted by *T. harzianum* EM0925 accounted for 3.23% of the total quantified CAZymes (Figure 2c). Moreover, the main glycosidases (glucosidases, xylosidases, arabinofuranosidases, and mannosidases) accounted for 17.2% of the total extracellular degradation enzymes secreted by *T. harzianum* EM0925 (Figure 3).

### 2.4. Properties of Enzyme Cocktail EM0925

The optimum temperatures of filter paper degrading enzymes and endoglucanases in the enzyme cocktail secreted by *T. harzianum* EM0925 were 60 °C, and that of xylanases were 50 °C. Filter paper activities and endoglucananse activities were 98.94% and 85.38% of their highest activities at 50 °C, respectively. When *T. harzianum* EM0925 enzymes were incubated at 50 °C for 1 h, the endoglucanases, total cellulases and xylanases still maintained 100%, 83.22%, and 85.72% relative activity, respectively.

**Figure 4:**
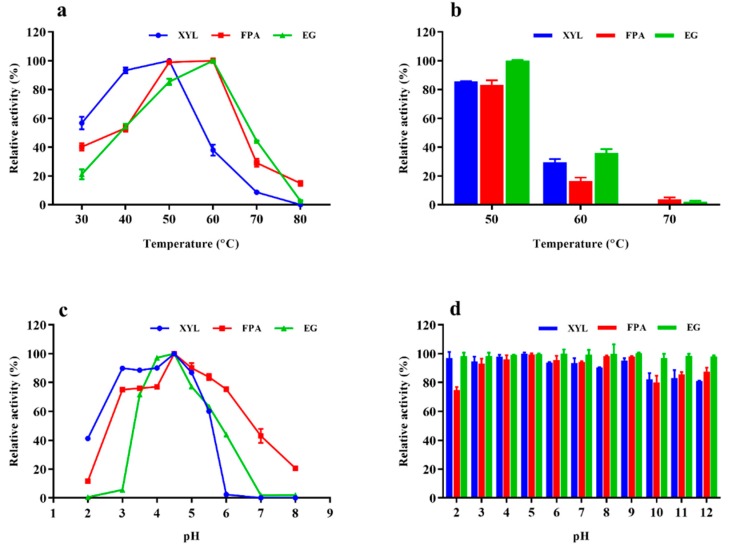
Characterization of FPA, CMCase, and xylanase of *T. harzianum* EM0925 enzyme cocktail. (**a**) The effect of temperatrure on FPA, CMCase, and xylanase of *T. harzianum* EM0925 enzyme cocktail; (**b**) thermostability of FPA, CMCase, and xylanase of *T. harzianum* EM0925 enzyme cocktail; (**c**) the effect of pH on FPA, CMCase, and xylanase of *T. harzianum* EM0925 enzyme cocktail; (**d**) pH stability of FPA, CMCase, and xylanase of *T. harzianum* EM0925 enzyme cocktail. pH stability was determined by measuring the residual activity after incubation of *T. harzianum* EM0925 enzyme cocktail in different buffers for 1 h. The relative activity was determined, and the maximum activity was defined as 100% (**a**,**c**). The initial activity of *T. harzianum* EM0925 enzyme cocktail not pre-incubated in different buffers was defined as 100% (**b**,**d**). Values are the mean of three replicates ± SD.

The optimal pH of total cellulases, endoglucanases and xylanases was 4.5. The activity was above 75% of the highest activity for total cellulase at pH 3.0–6.0, above 70% for endoglucanase at pH 3.5–5.0, and above 90% for xylanase at pH 2.5–5.0. In addition, favorable pH stability was also observed for the enzymes: 75% total cellulase activity was maintained after 1 h of incubation at pH-values between 2.0 and 12.0, in which 90% activity was maintained at pH 3.0 to 9.0. In addition, about 98% endoglucanase activity and 80% xylanase activity were maintained at the pH range of 2.0–12.0 after 1 h incubation, which 95% xylanase activity was maintained at the pH range of 2.0–9.0 (Figure 4).

### 2.5. Hydrolysis of Lignocellulosic Biomass by Enzyme Cocktail EM0925

**Figure 5:**
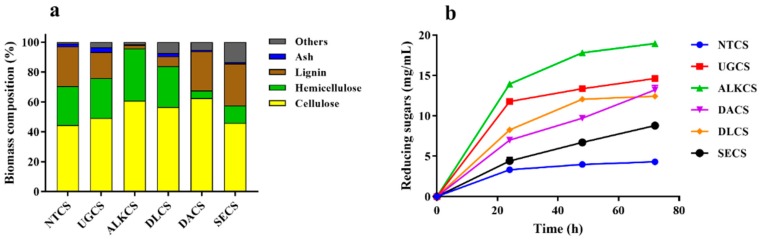
Reducing sugar yield from differently pre-treated fractions of corn stover by *T. harzianum* EM0925 enzyme cocktail. (**a**) Chemical compositon of pretreated corn stover. NTCS, matural corn stover without pretreatment; UGCS, ultrafine grinding corn stover; ALKCS, sodium hydroxide treated corn stover; DLCS, sodium chlorite treated corn stover; DACS, dilute acid treated corn stover; SECS, stream explosion treated corn stover. (**b**) Release of reducing sugars from differently pre-treated fractions of corn stover.

The structural carbohydrate and lignin contents of differently pre-treated fractions of corn stover were determined (Figure 5a). As expected, chlorite/acetic acid and NaOH pretreatment resulted a significant decrease in lignin content: only 2% of the initial lignin content remained in ALKCS, whereas the relative cellulose content was highly increased up to 60.7%, which was 1.4-fold of that in NTCS. Dilute acid and steam explosion treatment significantly decreased the content of hemicellulose, which decreased from 26.1% to 5% in DACS, and the highest cellulose content (62.5%) was observed at the same time (Figure 5a). At the enzyme dosage of 5 mg proteins/g substrate, hydrolysis with EM0925 generated 14.62, 19.00, 13.22, 12.43, and 8.83 mg reducing sugars per 20 mg of ultrafine grinding corn stover (UGCS), sodium hydroxide treated corn stover (ALKCS), sodium chlorite treated corn stover (DLCS), dilute acid treated corn stover (DACS), and stream explosion treated corn stover (SECS) after 72 h reaction, respectively. In contrast, only 4.31 mg reducing sugars were released from 20 mg of NTCS. Reducing sugar yield of UGCS and ALKCS was 96.6% and 100%, respectively. Furthermore, rapid saccharification was observed with these two substrates, which resulted in a sugar yield of 81.9% and 74.2% after 24 h of hydrolysis (Figure 5b).

**Figure 6:**
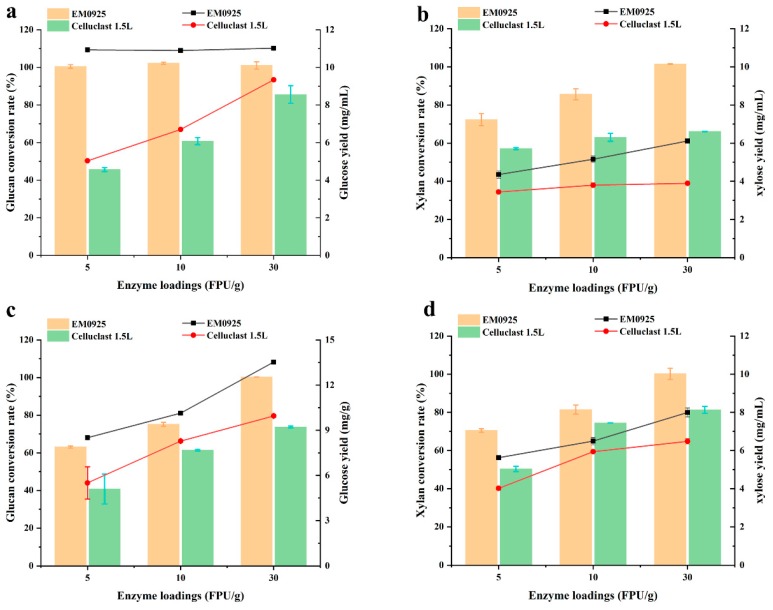
Conversion of glucan and xylan and sugar yield from UGCS and ALKCS by commercial enzyme preparation and *T. harzianum* EM0925 enzyme cocktails. (**a**) Glucan conversion and glucose yield from UGCS by commercial and *T. harzianum* EM0925 enzyme cocktails; (**b**) xylan conversion and xylose yield from UGCS by commercial and *T. harzianum* EM0925 enzyme cocktails; (**c**) glucan conversion and glucose yield from ALKCS by commercial and *T. harzianum* EM0925 enzyme cocktails; (**d**) xylan conversion and xylose yield from ALKCS by commercial and *T. harzianum* EM0925 enzyme cocktails. Celluclast 1.5 L, commercial cellulase preparation; UGCS, ultrafine grinding corn stover; ALKCS, sodium hydroxide treated corn stover.

To compare the saccharification efficiency of EM0925 with those of two commercial enzyme preparations, an enzyme loading of 5, 10, and 30 FPU/g substrate was selected as the low, moderate and high enzyme dosage, respectively. Glucose and xylose yields from UGCS and ALKCS were measured after 72 h of hydrolysis reaction. Cellulose of UGCS was completely converted to glucose by EM0925 at low enzyme dosage, and a xylan conversion of 72.35% was obtained simultaneously. Meanwhile, only 45.68% of the cellulose and 57.15% of the xylan were converted by Celluclast 1.5L. The xylan conversion gradually increased with increasing enzyme dosages, xylose yield was 100% when 30 FPU EM0925 enzymes/g substrate was used. Meanwhile, only 85.59% of the cellulose and 66.18% of the xylan were converted by Celluclast 1.5L even at the highest enzyme dosage (Figure 6a,b). When ALKCS was used as substrate, 63.08% cellulose conversion and 70.47% xylan conversion were obtained at low dosage of EM0925, which were 1.55 and 1.40-fold of the same conversions by Celluclast 1.5L. The yields of glucose and xylose were all gradually improved with increasing enzyme dosages of the two preparations. 100% glucose and xylose were released when 30 FPU/g EM0925 was used, and only 73.78% of the cellulose and 81.28% of the xylan were converted by Celluclast 1.5L at the highest enzyme dosage (Figure 6c,d).

When polysaccharides were completely converted to monosaccharides, 11.20 and 13.48 mg glucose were obtained from 20 mg of UGCS and ALKCS, respectively, while 6.11 and 7.98 mg pentose were released simultaneously. Monosaccharides yield was 865.5 mg/g of UGCS and 1073 mg/g of ALKCS, which was 1.29 and 1.31-fold of the highest monosaccharide yield obtained by Celluclast 1.5 L. In addition, the highest sugar yield from UGCS was obtained when EM0925 was applied at the dosage corresponding to 1/6 of the Celluclast 1.5 L dose (Figure 6a,b). These saccharification yields are among the highest yields reported in literature [29,30,31,32,33,34] (Table S1).

## 3. Discussion

Saprophytic fungi secrete a series of enzymes to degrade the plant cell walls in order to obtain nutrition for their growth. The main drawback of biofuels and other value-added bio-products generated from biomass are the high cost and the low saccharification efficiency of lignocellulolytic enzymes. In addition, the global market for industrial enzymes was expected to increase from nearly $5.0 billion in 2016 to $6.3 billion in 2021. Hence, lignocellulolytic enzymes which can be produced at low-cost and are capable of producing high yields of fermentable sugars require investigation. Using the agricultural residue corn stover as the sole carbon source, *T. harzianum* EM0925 secreted a quantitatively balanced enzyme system, which provides a low-cost enzyme preparation for biomass hydrolysis. High levels of endoglucanases and β-glucosidases could be secreted by *T. harzianum*, which has a high potential for application in industrial settings. According to Li et al. [12], the filter paper activity of the enzyme cocktail secreted by *T. harzianum* K223452 (CGMCC 17199) was 1.62 U/mL using sugarcane bagasse as the substrate supplied with 20 g/L glucose as carbon source. We observed a comparable activity for *T. harzianum* EM0925 when grown on corn stover without supplementation. These facts illustrate the possibility to develop low-cost degradation enzyme cocktails with high efficiency [2,26]. The high levels of lignocellulolytic enzymes and rapid enzyme production capacity of *T. harzianum* EM0925 made it a suitable candidate for enzyme cocktail production, since most of the previous studies on developing cost-effective cell wall degrading enzymes have focused on improving the activity of cellulase by adopting genetic operation and screening natural strains so as to obtain high yield of glucose [4,35]. Meng et al. [36] overexpressed the endoglucanase gene in *T. reesei* Rut-C30, which showed 90.0% and 132.7% increases in the activities of total cellulases and endoglucanases under flask culture conditions, respectively. Furthermore, hemicellulose is the second most abundant component of lignocellulose and is mainly composed of xylan, mannan, and xyloglucan. Hemicelluloses can be hydrolyzed into pentoses (xylose and arabinose) and hexoses (glucose, mannose and galactose) at high yield which is important for bio-refinery applications [37,38]. Besides the enzymes degrading the main chains of the structural polysaccharides in plant cell walls, various glycosidases have been found to be essential for obtaining fermentable sugars from lignocellulose [39]. Glucosidase, xylosidase, and arabinofuranosidase played key roles in the production of glucose, xylose, and arabinose, respectively. In addition, mannosidase, galactosidase, and esterase were also necessary to deconstruct the complex lignocellulose [40]. It was reported that *T. harzianum* LZ117 could secrete a more complete enzyme cocktail than *T. reesei*. This strain produced 29.24 U/mg xylanase after 144 h of fermentation, but its CMCase, *p*NPCase, and *p*NPGase were only 4.30, 0.19, and 1.81 U/mg, respectively, much lower than the other cellulase producers [12]. In our present study, CMCase, *p*NPCase, and *p*NPGase of EM0925 enzyme cocktail was 11.20, 33.76, and 84.97 U/mg, respectively. In addition, high levels of *p*NPXase and *p*NPAFase activities up to 203.60 and 11.72 U/mg were detected. Specific activities of glycosidases of EM0925 was the highest compared to the two extensively used inartificial enzyme cocktails under the conditions tested (Table 1). When using natural lignocellulose substrate as the sole carbon source, *T. harzianum* that secret cellulase and hemicellulase simultaneously, especially high levels of glycosidases has not been reported [27]. Therefore, the patterns of enzyme activities observed for *T. harzianum* EM0925 highlight its potential as efficient enzyme producer for industrial applications. 

Different induction conditions have been shown to greatly influence the secretion of cellulolytic enzymes by fungi as revealed by Filho et al. [20], at the transcriptional level. In the transcriptome of *T. harzianum*, the GH7 endoglucanase gene was the highest expressed one when grown on cellulose, while expression level of GH10 xylanase was significantly increased when grown on the natural bagasse [20]. Secretome analysis of *T. harzianum* EM0925 demonstrated the multiple functions and the synergistic effects of the lignocellulolytic enzymes. The balanced abundance of three kinds of cellulases play a crucial role for their synergistic degradation effects [41]. The abundant endoglucanases increased the frequency for randomly degrading the main chain of cellulose, which provided more access points for cellobiohydrolases. The high abundance of glucosidases could quickly remove the cellobiose and release glucose efficiently. In addition, much higher abundance of glycosidases responsible for side chain degradation could also effectively release series of fermentable sugars from complex polysaccharides [42]. A total of 44 glycosidases among the 81 CAZymes were found in the secretome of EM0925, with GH3, GH5, GH11, GH30, and GH55 as the dominant components, which may play crucial role in lignocellulose degradation (Figure S1). Vanessa et al. [43] also reported that the glycoside hydrolases were the most abundant class of proteins secreted by *Trichoderma harzianum* IOC 3844, which represent 67% of the total proteins; however, pretreated (partially delignified) cellulolignin from sugarcane bagasse was used in their study as the inducer for lignocellulosic enzyme secretion. In addition, the most abundant groups in the secretome of *T. harzianum* IOC 3844 were GH3 (17%), GH5 (11%), GH 7 (10%), GH6 (6%), and GH55 (6%), which was slightly different from those verified for *T. harzianum* EM0925 in our present study and demonstrated the possible effects of the secretome.

For lignocellulose degradation, the amount of cellulases is not the most critical factor affecting sugar yield, since the synergistic effects between the cellulases and hemicellulases and between the enzymes for main- and side-chain degrading enzymes of the polysaccharides complex might be more important. Therefore, the high conversion efficiency of the secretome of *T. harzianum* EM0925 might be achieved from its numerously and quantitatively balanced cellulases and hemicellulases. Previously, partial replacement of cellulases with auxiliary enzymes has reduced the required amount of commercial cellulases to achieve high hydrolysis yields [11,44]. Yang et al. [45] reported a degree of synergy of 1.35 for the hydrolysis of delignified corn stover when studying the synergistic effect between a commercial enzyme preparation and a bifunctional enzyme consisting of an acetyl xylan esterase and a α-l-arabinofuranosidase. For glucan conversion of delignified corn stover, Zhu et al. [46] found a synergy of 1.96 between a hemicellulase preparation EMSD5 (including xylanases, β-xylosidases, α-l-arabinofuranosidases, α-glucuronidases, and acetyl xylan esterases) and the commercial cellulase from *T. reesei*. These findings revealed the significant role of glycosidases for efficient lignocellulose degradation. Moreover, 9 carbohydrate binding modules (CBMs) were also observed, which could help hydrolyze substrate more effectively [46]. In *T. harzianum* EM0925 enzyme enzyme cocktail, a LPMO with abundance of 3.23% was found. It was discovered that LPMOs promote degradation of the most recalcitrant crystalline cellulose by carrying out oxidative cleavage of polysaccharides [47]. Several studies showed synergetic effects between LPMOs and the lignocellulolytic enzymes during the saccharification process of cellulose and hemicellulose components [9,47]. All CAZymes and non-hydrolyzed protein detected in the secreted proteins of *T. harzianum* EM0925 are important for fermentable sugars production. In addition, some glycoside hydrolases such as GH30 xylanase and GH26 mannase secreted by *T. harzianum* EM0925 have been rarely studied in *Trichoderma*. Synergy between cellulolytic enzymes and these enzymes from *Acremonium alcalophilum* and *Aspergillus nidulans* has been described, which deserves more attention in the future [48,49]. The currently reported lignocellulosic degrading bacteria and fungi with industrial application prospects presented distinct optimal conditions for different enzymes even in the same induced enzyme system, and the stabilities against temperature and pH were also different, so it is necessary to determine the optimal conditions for enzymatic hydrolysis and saccharification with overall consideration [1,50,51]. In the present study, similar optimal temperature and pH for various functional enzymes in *T. harzianum* EM0925 enzyme cocktail were observed, which gave rise to better synergistic effects in efficient deconstruction of lignocellulose complex. Pretreatments could make the macroscopic and microscopic deconstruction and change chemical composition of lignocellulose, which improve the accessibility of enzymes and facilitate release of fermentable sugars. Among many pretreatment methods, dilute alkali treatment is widely used because of its remarkable effect, and physical crushing pretreatment without any pollution has been widely used as well [52]. 

When EM0925 was used, reducing sugar yields of 96.6% and 100% were obtained from UGCS and ALKCS, respectively, after 72 h of saccharification. The efficacy after 24 h of saccharification was 81.9% and 74.2%, respectively. (Figure 5b). So, the enzyme cocktail EM0925 was much more effective than the current commercial products under the conditions tested, since only 46.9% sugar yield was acquired from UGCS by Celluclast 1.5 L after 72 h hydrolysis [33] (Table S1). In general, saccharification efficiency of lignocellulose biomass by EM0925 was better than that in any other literature reported under the conditions tested [29,30,31,32,33,34]. To be specific, in our study, the maximum 85.59% glucose yield and 66.18% xylose yield were obtained from UGCS hydrolyzed with 30 FPU/g of Celluclast 1.5 L used; while 100% glucose yield and 100% xylose yield were obtained from UGCS and ALKCS with the same enzyme dosage of *T. harzianum* EM0925. To some extent, the glucose and xylose yields by EM0925 were lower than the total sugar yields when the same substrates were used, which indicated that not only monosaccharides but also oligosaccharides were obtained from UGCS and ALKCS. From these results, it could be estimated that various auxiliary enzyme including hemicellulase and amylase played important role to completely and effectively deconstruct the complex lignocellulose substrates [11].

Some accessory enzymes that assist biomass degradation could be used to improve the recovery of fermentable sugars for use in a biorefinery setting in order to improve total utilization of biomass. Supplementation of a commercial enzyme preparation with 30% crude enzyme complex from *Aspergillus oryzae* P21C3 increased the conversion of cellulose derived from pretreated sugarcane bagasse by 36%, which demonstrated the potential to use the supplementary enzymes in the total lignocellulose degradation, although 51.2% of cellulose conversion and 78.1% of xylan conversion were obtained [53]. With supplementary of β-glucosidase produced by *Aspergillus niger* and endoglucanase produced by *Talaromyces emersonii* to the currently used commercial enzyme cocktails, greater saccharification of alkaline-pretreated bagasse (87% glucose yield and 94% of xylose yield) was obtained, but its enzyme dosage was as high as 500 FPU/g substrate [54]. In the present study, only enzyme dosage of 5 mg EM0925/g substrate (13.85 FPU/g) was required for complete saccharification of lignocellulose, which provided sufficient sugars for the downstream fermentation of downstream products. Therefore, EM0925 presented great potential in industrial applications. To the best of our knowledge, it was the first example that enzyme cocktail from *T. harzianum* completely and simultaneously converted cellulose and xylan in natural biomaterials into fermentable sugars at the same time. To date, induction and saccharification mechanisms of lignocellulosic enzymes secreted by *T. harzianum* are still unknown. Differences in the composition of the *T. harzianum* secretome in response to different substrates was reported, which revealed the diversity of the fungus enzymatic toolbox [55]. Nevertheless, enzyme cocktails with excellent efficiency of complete saccharification of lignocellulose should be tailor-made, and degradation mechanisms remain to be elucidated in more detail. The expression of total extracellular proteins of *T. harzianum* EM0925 also could be optimized at the molecular level, in order to further expand the application scope of *T. harzianum* EM0925. 

## 4. Materials and Methods

### 4.1. Strain and Culture Conditions

*Thrichoderma harzianum* EM0925 was isolated and screened for activities of lignocellulolytic enzyme in our laboratory previously. For enzyme production, the strain was incubated on potato agar for 3 days at 30 °C, then, 5 slices with diameter 0.5 cm of the agar plate were inoculated into each flask containing 200 mL Mandel’s salt solution (4.0 KH_2_PO_4_, 2.8 (NH_4_)_2_O_4_, 0.6 MgSO_4_·7H_2_O, 0.01 FeSO_4_·7H_2_O, 0.003 MnSO_4_·H_2_O, 0.003 ZnSO_4_·7H_2_O, 0.004 CoCl_2_·6H_2_O, and 2.0 peptone, in g·L^−1^) supplemented with 2% (*w/v*) lignocellulose substrate at 30 °C [15].

### 4.2. Preparation of Enzyme Cocktail EM0925

Different carbon sources were used to induce the enzyme production of *T. harzianum* EM0925. Lignocellulosic enzyme activities in the culture solution were measured each day, the optimal incubation time was estimated according to the filter paper activity (FPA), and the activities of endoglucanase (EG) and xylanase (XYL) [15]. Ammonium sulfate precipitation with a final concentration of 85% (w/v) was used to prepare the enzyme cocktails. After incubation at 4 °C for 4 h, the precipitate fraction containing the secreted enzymes was collected by centrifugation at 10,000× *g* for 20 min at 4 °C and then dissolved in sodium acetate buffer (50 mM, pH 5.0) and filtered through a 0.45 μm filter membrane (Millipore, Bedford, MA, USA). Proteins in the supe rnatant were dialyzed against sodium acetate buffer (50 mM, pH 5.0) and then freeze-dried. Commercial cellulase enzyme C 9748 derived from *Trichoderma longibrachiatum* (C 9748, Sigma Aldrich, St. Louis, MO, USA) and Celluclast 1.5 L from *Trichoderma reesei* ATCC 26921 (Sigma, USA, Novozyme products) were used as reference. The protein content of the enzyme cocktail was determined by a Bradford protein assay regent (Bio-rad, Hercules, CA, USA) [12].

### 4.3. Enzymatic Assays

FPA was measured according to the method reported by Urbánszki et al. [56]. EG and XYL were measured with carboxymethyl cellulose (CMC-Na, TCI, Tokyo, Japan) and Beechwood xylan (Sigma, USA) as substrates, respectively. Briefly, to measure the xylanase activity, an aliquot of 100 μL diluted crude enzyme was mixed with 100 μL 1% (*w*/*v*) of xylan. After 15 min of incubation at 40 °C, 150 μL of the 3,5-dinitrosalicylic acid (DNS) reagent was added. For CMCase activity measurement, an aliquot of 50 μL of diluted crude enzyme was mixed with 150 μL of 0.5% carboxymethyl cellulose. After incubation at 50 °C for 30 min, 50 μL of 1 M NaOH and the DNS reagent were sequentially added. After boiling the mixture at 100 °C for 5 min, it was cooled in cold water and the absorbance was read at 540 nm. Activities were calculated with glucose or xylose as the standard. One unit of enzyme activity was defined as the amount of enzyme catalyzing the release of 1 μmol of reducing sugars in 1 min from the substrate under the above conditions [57]. The cellobiohydrolase (CBH), β-d-glucosidase (βG), β-xylosidase (βX) and α-l-arabinofuranosidase (ABF) activities were determined according to the protocol of Zhu et al. [46] with minor modification: using *p*-nitrophenyl-β-d-cellobioside (*p*NPC), *p*-nitrophenyl-β-glucopyranoside (*p*NPG), *p*-nitrophenyl-β-d-xylopyranoside (*p*NPX), and *p*-nitrophenyl-α-l-arabinofuranoside (*p*NPAF) as substrate, respectively. The reaction mixture contained 50 μL of enzyme, 50 μL of 50 mM sodium acetate buffer (pH 5.0), and 50 μL of 10 mM substrate solution. After incubation at 50 °C for 10 min, the reaction was terminated by adding 50 μL of 1 M Na_2_CO_3_ and the absorbance of released *p*-nitrophenol was measured at 405 nm. Activities were calculated using p-nitrophenol as the standard. One unit of enzyme activity was defined as the amount of enzyme that produced 1 μmol of *p*NP in 1 min from the substrate under the above conditions. In addition, β-mannase activity (Man) was determined by the DNS method using mannose as the standard according to the methods of Li et al. [38]. The amylolytic activity (Amy) was assayed by the DNS method using maltose as the standard as reported by He et al. [58].

To determine the optimal temperature for FPA, EG, and XYL of the enzyme cocktail from *T. harzianum* EM0925, assays were performed in the temperature range from 30–70 °C at an interval of 10 °C. The optimal pH values for cellulases and hemicellulases were investigated at the optimal temperature in buffers ranging from pH 2–8 at intervals of 0.5 pH units, using 0.05 M different buffers: glycine-HCl (pH 2.0–3.0), sodium acetate (pH 4.0–5.0), and citric-Na_2_HPO_4_ (pH 6.0–8.0). Thermal stability of EM0925 was assessed by measuring the residual activity in the optimal conditions after incubation of the cocktail at different temperature (50, 60, and 70 °C) for 1 h. A dose of 1.0 mg/mL of enzyme cocktail was pre-incubated in 0.05 M sodium acetate, enzyme cocktail without pre-incubated was used as the control. The stabilities of various enzymes at different pH values were determined by measuring the residual activities in standard conditions after the enzyme incubation in 0.05 M buffers of different pH (pH 2.0, 3.0, 4.0, 5.0, 6.0, 7.0, 8.0, 9.0, 10.0, 11.0, and 12.0) at room temperature for 1 h. The buffers used were glycine-HCl (pH 2.0, 3.0), sodium acetate (pH 4.0, 5.0), citric-Na_2_HPO_4_ (pH 6.0–8.0) and glycine-NaOH (pH 9.0–12.0). One mg/mL of enzyme cocktail was pre-incubated in 0.05 M of the corresponding buffers, buffers, and enzyme cocktails without pre-incubated was used as the control [45].

### 4.4. Biomass Pretreatment and Component Analysis

The mature corn stover was collected from Shandong Province of China, and the other biomasses were available in our laboratory. The dried biomasses were ground and passed through a 50 mesh sieve before drying at 50 °C to a constant weight. Un-treated corn stover (NTCS) was subjected to different pretreatments as follows. Ultrafine grinding pretreatment corn stover (UGCS) was prepared by mixing NTCS and ZrO_2_ balls (6–10 nm diameter) with a ratio of 1:2 and grinding for 0.5 h in a CJM-SY-B ultrafine vibration ball mill (Taiji Ring Nano Products Co., Hebei, China) [33]. Alkali-treated corn stover (ALKCS) was prepared by mixing the biomass and 1% (w/v) of NaOH at a solid-liquid ratio of 1: 10 and autoclaving the mixture at 121 °C for 1 h [59]. Sodium chlorite/acetic acid delignificated corn stover (DLCS) was obtained according to the protocol of Hu et al. [44]. Dilute acid pretreated corn stover (DACS) was treated with dilute acid according to the procedure of Ji et al. [59]. The steam explosion pretreated corn stover (SECS) was obtained by steam exploding the substrate according to the procedure of Liu et al. [57]. All slurries were filtered and washed until the pH reached 5.0 prior to use.

The structural carbohydrate and lignin contents of different pretreated biomasses were determined according to the laboratory analytical procedure of the National Renewable Energy Laboratory [60]. In brief, a sample of 0.5 g dry biomass was hydrolyzed at 30 °C with 3.0 mL H_2_SO_4_ (72%, w/v) for 1 h. Then 84 mL deionized water was added and a second hydrolysis was carried out in the autoclave at 121 °C for 1 h. All slurries were filtered and washed until reaching neutral pH. The glucose and xylose concentrations in the filter liquor were determined by HPLC. Acid-insoluble lignin was determined by subtracting the ash content from the solid residue dried at 60 °C. The ash content was determined by heating the solid residue at 575 °C for 12 h. The weight percentages of cellulose, xylan, arabinan, and lignin were calculated according to the method described previously [46]. 

### 4.5. Enzymatic Hydrolysis

Hydrolysis of all biomasses was conducted in 50 mM sodium citrate buffer (pH 4.5) in a 1 mL volume system containing 2% (*w/v*) substrate. The reaction was conducted in a 37 °C air bath with shaking at 200 rpm. Varying amounts (5 mg protein/g substrate and 5, 10, 30 FPU/g substrate) of enzyme loading were used for hydrolysis reactions. Samples were taken after 8 h, 12 h, 24 h, 48 h and 72 h of hydrolysis, and then heated at 100 °C for 10 min to terminate the reaction. Supernatants were collected by centrifugation at 10,000× *g* for 10 min. The content of total reducing sugars was determined by the DNS method. Monosaccharides in samples were determined by HPLC. Treatments containing substrate or enzyme alone were used as blank controls [15].

### 4.6. Extracellular Quantitative Proteome Analysis

The freeze-dried enzyme preparation of *T. harzianum* EM0925 dissolved in deionized water was separated by 12% (*w/v*) sodium dodecyl sulphate-poly-acrylamide gel electrophoresis (SDS-PAGE) at 120 V. The gel bands were excised and subjected to tryptic digestion and separated. The resulting peptides were reconstituted in 0.1% formic acid and then label-free quantitative proteomics identification by LC-MS/MS was performed. MaxQuant (version 1.4.1.2, https://www.maxquant.org/maxquant/) was used as the search engine in order to identify and quantify the extracellular proteins of *T. harzianum* EM0925. Raw data were preprocessed with Mascot Distiller 2.5 (Matrix Science, Boston, MA, USA) for peak picking, and resulting peaks were searched against the UniProt database (https://www.uniprot.org/) for *T. harzianum* strain CBS 226.95. The remaining parameter “intensity-based absolute quantification (iBAQ)” option was enabled. Only proteins identified with at least two peptides were considered for label-free quantification. Calculation of the protein label free quantification (LFQ) intensity was based on unique peptides using the built-in label-free quantification algorithm. The identified proteins were annotated using the Pfarm (https://pfam.xfam.org/search#tabview=tab1) and dbCAN database (http://bcb.unl.edu/dbCAN2/). Relative quantifications of extracellular proteins of *T. harzianum* EM0925 were analyzed. Three technical replicates were included in all the analyses [42,61].

## 5. Conclusions

Under induction of lignocellulosic biomass, *T. harzianum* EM0925 could rapidly secrete glycoside hydrolases, including numerically and quantitatively balanced cellulases and hemicellulases and especially high levels of glycosidases. *T. harzianum* EM0925 enzyme cocktail presented significantly higher enzyme activities than the commercial preparations under the conditions tested, and released 100% of glucose and xylose from UGCS and ALKCS simultaneously. These findings clearly indicate that *T. harzianum* EM0925 enzyme cocktail has great potential for industrial applications.

## Figures and Tables

**Table 1:** Cellulase and hemicellulase activities of EM0925 and commercial enzyme preparations (specific activities, U/mg protein).

Cocktail	FPA	EG	CBH	βG	Xyl	βX	Ara	Man	Amy
C 9748	1.60 ± 0.20 ^a^	19.70 ± 2.90 ^a^	52.20 ± 1.90 ^a^	29.0 ± 2.70 ^a^	26.60 ± 3.20 ^a^	34.80 ± 1.40 ^a^	0.03 ± 0.00 ^a^	0.71 ± 0.00 ^a^	0.30 ± 1.00 ^a^
Celluclast 1.5 L	1.90 ± 0.02 ^a^	8.33 ± 0.18 ^b^	32.70 ± 1.80 ^b^	0.27 ± 0.002 ^b^	10.80 ± 0.14 ^b^	0.61 ± 0.02 ^b^	0.31 ± 0.00 ^b^	3.90 ± 0.11 ^b^	ND
EM0925	2.77 ± 0.08 ^b^	11.20 ± 0.13 ^c^	33.76 ± 2.40 ^b^	84.97 ± 0.62 ^c^	314.70 ± 3.23 ^c^	203.60 ± 18.60 ^c^	11.72 ± 0.33 ^c^	6.70 ± 0.12 ^c^	3.92 ± 0.02 ^b^

FPA, filter paper activity; EG, CMCase activity; CBH, cellobiohydrolase activity; βG, β-glucosidase activity; Xyl, xylanase activity; βX, β-xylosidase activity; Ara, arabinofuranosidase activity; Man, mannase activity; Amy, amylase activity; C 9748, commercial cellulase preparation from Sigma; Celluclast 1.5 L, commercial cellulase preparation from Novozyme; EM0925, enzyme cocktail of *T. harzianum* EM0925; ND, not detected. ^a, b, c^ statistical significance is indicated by different letters in table as assessed by ANOVA (*p* < 0.05).

## Supplementary Materials

The following are available online at https://www.mdpi.com/1422-0067/21/2/371/s1, Table S1: A comparison of different previous reported enzyme cocktails for lignocellulose saccharification. Figure S1: Distribution of glycoside hydrolase family in proteome of *T. harzianum* EM0925.

## Abbreviations

CAZy	Carbohydrate active enzymes database
CBH	Cellobiohydrolase
CBM	Carbohydrate-binding module
EG	Endoglucanase
GH	Glycoside hydrolase
LPMO	Lytic polysaccharide monooxygenase
HPLC	High-performance liquid chromatography
FPA	Filter paper activity
CMC	Carboxymethylcellulose
βG	β-glucosidase
Xyl	Xylanase
βX	β-xylosidase
Ara	Arabinofuranosidase
Man	Mannase
Amy	Amylase
LC-MS/MS	Liquid chromatography-tandem mass spectrometry
NTCS	Non-pretreated corn stover
UGCS	Ultrafine grinding pretreated corn stover
ALKCS	Alkali pretreated corn stover
DACS	Dilute acid pretreated corn stover
DLCS	Sodium chlorite/acetic acid delignificated corn stover
SECS	Steam explosion pretreated corn stover
*p*NPC	*p*-nitrophenyl-β-d-cellobioside
*p*NPG	*p*-nitrophenyl-β-d-glucoside
*p*NPX	*p*-nitrophenyl-β-d-xyloside
*p*NPAF	*p*-nitrophenyl-α-l-arabinofuranoside
iBAQ	Intensity-based absolute quantification
LFQ	Label free quantification
