# Supplementary material for: Low-Cost Cellulase-Hemicellulase Mixture Secreted by Trichoderma harzianum EM0925 with Complete Saccharification Efficacy of Lignocellulose
Source: Int J Mol Sci. 2020 Jan 7;21(2):371. doi: 10.3390/ijms21020371 (PMC7014229; doi:10.3390/ijms21020371)
Supplement: Supplementary file 1 [file ijms-21-00371-s001.pdf]

Supplementary Materials:

Table S1. A comparison of different previous reported enzyme cocktails for lignocellulose saccharification.

Enzyme Cocktails	Substrate/Pretreatment	Enzyme Loading	Time	Saccharification Yield	Reference
Spezyme CP	Switchgrass Ball-milled	26 mg/g	72 h	100% glucose	Boussaid et al., [29]
Spezyme CP+Novozyme	Switchgrass Ionic liquid	109 mg/g	24 h	91% glucose	Li et al., [30]
Cellic CTec 2	Wheat straw Hot water	27 mg/g	96 h	95% glucose	Herbaut et al., [31]
Cellic CTec 2	Wheat straw Ionic liquid	27 mg/g	96 h	45% glucose	Herbaut et al., [31]
Cellic CTec 3	Spruce Sodium sulfite	10.5 mg/g	48 h	76% glucose	Chylenski et al., [32]
Celluclast 1.5L	Corn stover Ultrafine grinding	10 mg/g	72 h	49.6% glucose	Li et al., [33]
Spezyme CP+Novozyme 188+xylanase+pectinase	Prairie NaOH	25 mg/g	72 h	74% glucose 78% xylose	Sills et al., [34]
Celluclast 1.5L	Corn stover NaOH	15 mg/g	72 h	85.6% glucose 66.2% xylose	This study
EM0925	Corn stover NaOH	10.8 mg/g	72 h	100% glucose 100% xylose	This study
EM0925	Corn stover Ultrafine grinding	10.8 mg/g	72 h	100% glucose 100% xylose	This study

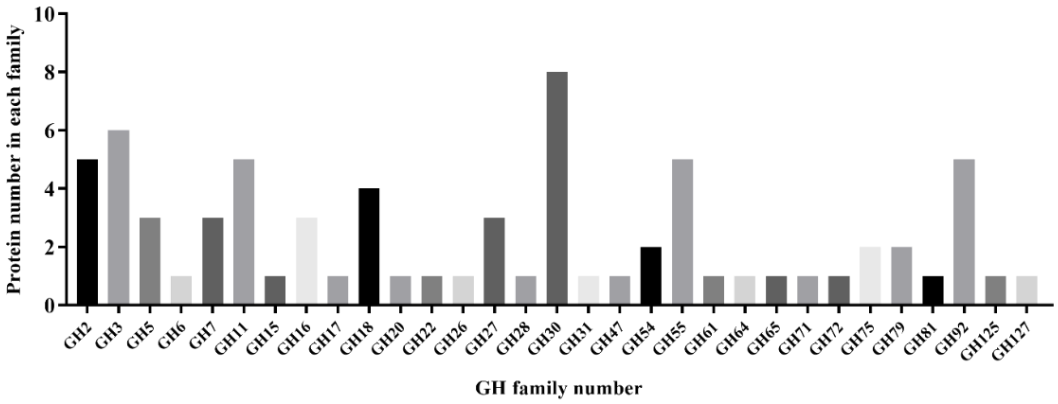

Figure S1. Distribution of glycoside hydrolase family in proteome of *T. harzianum* EM0925.
